# Topography of the Papillary Muscles in the Mitral Valve Complex and Their Relevance for Mitral Valve Function

**DOI:** 10.3390/jcdd12090348

**Published:** 2025-09-11

**Authors:** Alina-Jutta Van Laethem, Jens Figiel, Andreas H. Mahnken, Rabia Ramzan, Marc Irqsusi, Sebastian Vogt, Ardawan J. Rastan

**Affiliations:** 1 Heart and Cardiovascular Surgery, Philipps-University Marburg, 35043 Marburg, Germany; alivanlaethem@hotmail.de (A.-J.V.L.); ramzan@med.uni-marburg.de (R.R.); irqsusi@med.uni-marburg.de (M.I.); a.rastan@uk-gm.de (A.J.R.); 2 Diakonie-Hospital Wehrda, 35041 Marburg, Germany; j.figiel@dkh-wehrda.de; 3 Clinic for Diagnostic and Interventional Radiology, University Hospital Marburg, 35043 Marburg, Germany; mahnken@med.uni-marburg.de

**Keywords:** papillary muscles, mitral valve insufficiency, topographic anatomy

## Abstract

**Background:** The mitral valve apparatus is a complex system that requires sufficient function of all involved structures. Previous studies have demonstrated that ventricular remodeling can cause displacement of subannular structures, including the papillary muscles, which in turn promotes the development of mitral regurgitation. Furthermore, in such cases, annuloplasty alone is often insufficient to restore optimal valve function. Instead, additional reconstruction of the subannular apparatus is associated with improved clinical outcomes. Our study aimed to analyze the topography of the papillary muscles in the mitral valve complex and their relevance for mitral valve function. **Methods:** In 148 patients who underwent both cardiac computed tomography (CT) and echocardiography, the position of the papillary muscles within the left ventricle was assessed. CT scans were evaluated in end-diastolic four-chamber view, two-chamber view, and short-axis view. CT analysis involved determining the position of the papillary muscles based on a modified left ventricular segmentation scheme, which subdivided the original segments into “a” and “b” subsegments in a counterclockwise manner. Furthermore, the midventricular diameter, ventricular length, as well as the angle between the papillary muscle (PM) and the left ventricular wall, were measured. Comorbidities were assessed. The presence of mitral regurgitation (MR) and ejection fraction was determined based on echocardiographic data. Echocardiography was conducted either as part of initial cardiological assessments or during follow-up examinations. For detailed statistical analysis, the patients were divided into the following groups: control group, MR-only group, coronary heart disease (CHD)-only group, and combined CHD and MR subgroup. **Results:** Mitral regurgitation was significantly correlated with age (*p* < 0.001) and hypertension (*r* = 0.1900, *p* = 0.0208), and in the MR-only subgroup, additionally with atrial fibrillation (*r* = 0.2426, *p* = 0.0462). The length (*p* < 0.001) and internal diameter (*p* < 0.001) of the left ventricle were significantly larger in men than in women. Different positions of the papillary muscles were identified. Segment 7a was significantly correlated with MR in the combined CHD and MR subgroup. In normal-sized ventricles, patients with MR and papillary muscle in 12a (*p =* 0.0095) or 10a (*p =* 0.0460) showed a significantly larger angle than patients without MR (overall dataset). **Conclusions:** Assessment of papillary muscle position is essential in diagnosing mitral regurgitation and should guide the consideration of subannular repair during surgical treatment.

## 1. Introduction

Interaction between all structures of the mitral valve complex (annulus, leaflets, chordae tendineae, papillary muscles, and left ventricular wall) is essential for a sufficient geometry and function of the mitral valve complex [1]. Papillary muscles actively participate in the coordinated opening and closing of the mitral valve leaflets [2]. Optimal valve function requires correct angular positioning of the papillary muscles and connection to the mitral valve leaflets [3,4]. An altered position of the papillary muscles in the mitral valve apparatus—often secondary to ventricular remodeling—can cause leaflet tethering and impaired coaptation of the leaflets, resulting in MR [5]. Accordingly, preoperative assessment of the PM position has acquired clinical significance due to its central role in the pathophysiology of mitral valve regurgitation, as it can significantly influence both the appropriate reconstructive strategy and postoperative outcome [6,7]. In this context, cardiac CT has emerged as a high-resolution imaging modality that enables comprehensive visualization of the mitral valve apparatus, including precise determination of the papillary muscles in relation to the valve plane and throughout the entire left ventricle [8]. 

The first part of this study was to show a variation in the position of papillary muscles, and the second was to consider its relevance to mitral valve function.

## 2. Materials and Methods

A total of 473 patients underwent cardiac computed tomography (CT) between 2010 and 2016. All CT datasets were performed using a dual-source CT scanner (SOMATOM Definition, Siemens™, Forchheim, Germany). Exclusion criteria included post-resuscitation cases, scans with incomplete depiction of the target structure, and those with significant motion artifacts. Ultimately, 272 CT datasets were eligible for further analysis. Furthermore, all patients who did not receive additional echocardiography (Philips iE33™ 1815 Industrial Drive, Suite 100, Stockton, CA, USA) (transthoracic or transesophageal) were excluded. In the end, a total of 148 patients, 89 men and 59 women, who underwent both cardiac computed tomography and echocardiography for diagnostic purposes, were included in our study population. Indication for CT was a non-invasive work-up for coronary heart disease in patients with dyspnea, angina pectoris, arrhythmia, or abnormalities in stress echocardiogram. Echocardiography was conducted either as part of initial cardiological assessments or during follow-up examinations. Data were retrospectively analyzed.

**Figure 1:**
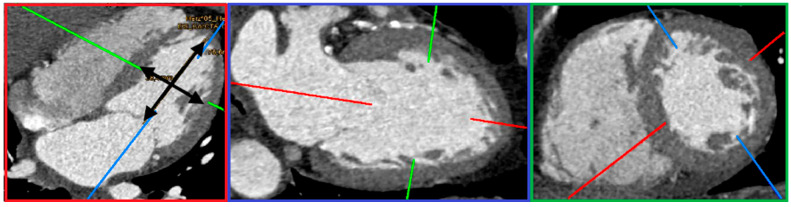
Multiplanar reconstruction in end-diastole. In the four-chamber view (red box), the right atrium and right ventricle, as well as the left atrium and left ventricle, are depicted. The black arrows indicate the measurements of ventricular length and midventricular diameter performed in our study. The two-chamber reconstruction (blue box) displays both the short (green line) and long axis (red line), with the long axis passing through the center of the mitral valve and the apex of the ventricle. In the green box, the reconstruction of the short axis (depicted by the green line in the other boxes) is shown. The short axis (green box) is defined as being perpendicular to the long axis (red line) (based on the approach of Stolzmann et al. [9]).

Data from cardiac computed tomography were visualized with Picture Archiving and Communication System (PACS) IMPAX EE (Agfa HealthCare N.V, Mortsel, Belgium). Measurement of the left ventricle included length, midventricular diameter, papillary muscles’ midventricular position, and the angle between the ventricular wall and each papillary muscle. CT scans were evaluated in end-diastolic four-chamber view, two-chamber view, and short-axis view (Figure 1), using the Multiplanar Reconstruction (MPR) Plugin in IMPAX EE cardiovascular 7.7 and 7.8 Doc. 001341 (www.agfa.com, Agfa HealthCare N.V. Mortsel, Belgium). Standardized angulations in two- and four-chamber view with one axis running through the apex and the center of the mitral valve were used. The two-chamber view shows the left ventricle and left atrium; in the four-chamber view, both ventricles and atria are displayed [9].

**Figure 2:**
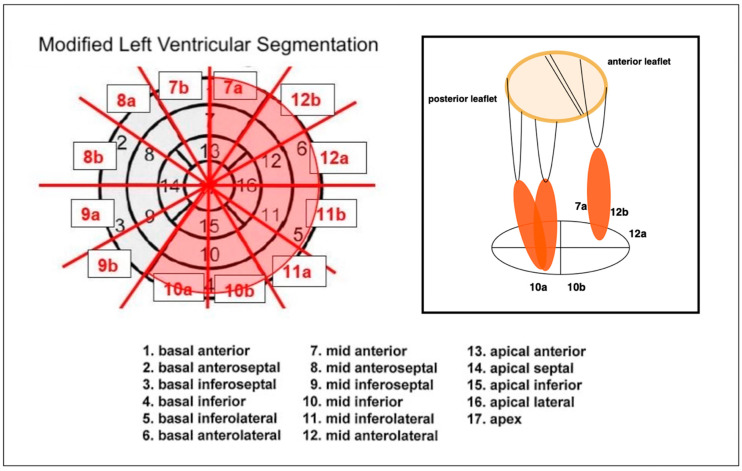
Modified left ventricular segmentation (original left ventricular segmentation by Cerqueira et al. [10]) and simplified illustration of the papillary muscles in anterolateral and posteromedial position. The red-highlighted areas within the segmentation scheme indicate the segments relevant for the determination of the papillary muscle position.

The position of the papillary muscles was determined according to the left ventricular segmentation scheme of the American Heart Association Scientific Statement [10]. We detected the positions of the papillary muscles in a midventricular short-axis view. Since the original left ventricular segmentation scheme encompasses relatively large regions, certain changes in the position of the papillary muscles may not be adequately captured. To enable a more detailed localization, the segments were further subdivided into sub-segments ‘a’ and ‘b’, in a counterclockwise manner (Figure 2). Both anterolateral and posteromedial papillary muscles were identified in each scan.

**Figure 3:**
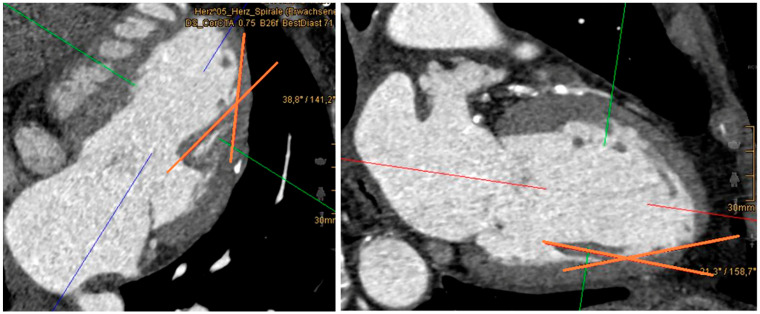
Measurement of the angle between the papillary muscle and left ventricular wall (red bold lines). The picture on the left shows the anterolateral papillary muscle, the picture on the right the posteromedial papillary muscle. In both measurements, one axis passes through the papillary muscle itself, while the other axis is defined as a tangent at the muscle’s point of origin.

Because of differences in the anatomical course of the papillary muscles, the angle between the left ventricular wall and each papillary muscle was measured. Therefore, papillary muscles were visualized in the two-chamber view. To calculate the angles, one axis was placed through the papillary muscle; the second axis was defined as a tangent to the ventricular attachment of the papillary muscle (Figure 3). Measurement of the posteromedial papillary muscle occasionally proved challenging due to its marked heterogeneity. In cases presenting with multiple muscle heads, the angle was determined at the thickest muscle head. Similarly, within the ventricular segmentation scheme, localization was based on the most dominant muscle head.

To prevent affection of the angles by ventricular dilation, we separated patients into two groups: those with normal-sized ventricles and those with dilated ventricles. Male ventricles with a diameter of more than 54 mm and female ventricles with a diameter of more than 52 mm were included in the group with dilated ventricles.

Echocardiographic data obtained by cardiologists independently and prior to our study were analyzed for the presence of mitral valve regurgitation. The severity of regurgitation was classified as ‘not hemodynamically relevant’, ‘mild’, ‘moderate’, or ‘severe’, with an additional category for ‘prolapse’. For statistical analysis, patients were categorized into two groups: “no mitral regurgitation” and “mitral regurgitation”. The latter group encompassed all patients with abnormal echocardiographic findings, irrespective of the severity of the regurgitation.

For a more detailed analysis of the data, patients were subdivided into the following subgroups:

(1) Control group (31 patients): This subgroup included all patients without mitral regurgitation (MR) or coronary heart disease (CHD). All individuals in this group had a normal ejection fraction (EF).
(2) MR-only subgroup (37 patients): Patients in this group had isolated MR without coexisting CHD. A reduced EF was observed in 5 of these patients.
(3) CHD-only subgroup (25 patients): Patients in this group had isolated CHD without MR. EF was reduced in 2 of these individuals.
(4) Combined CHD and MR subgroup (55 patients): These patients had both CHD and MR. EF was reduced in 18 of them.

The subdivision in the subgroups “MR-only” and “Combined CHD and MR” was based solely on the presence of mitral regurgitation in combination with either the presence or absence of coronary heart disease. Patients with MR but without any form of CHD were classified as “MR-only”, while those with both MR and CHD were included in the “combined CHD and MR” subgroup. Each pathological subgroup was compared with the control group for subgroup-specific statistical analysis. All subgroups were included in the overall dataset.

Additionally, comorbidities, including hypertension, diabetes mellitus, atrial fibrillation, and nicotine use, as well as current medication regimens were recorded. In 118 patients, height and weight were also determined. The body surface area (BSA) was calculated according to Mosteller’s formula.

## 3. Statistical Data Analysis

All data were analyzed with the free version of RStudio (Posit team (2023, Version 2023.12.0+369). RStudio: Integrated Development Environment for R. Posit Software, PBC, Boston, MA, USA). Differences between groups were tested with Pearson’s product moment correlation, Chi-square test, Fisher’s exact test, regression analysis, or Mann–Whitney U test without continuity correction. The significance level was determined with *p* < 0.05.

## 4. Results

Among 148 patients, 89 men and 59 women, 92 patients (62.16%) were affected by a pathology of the mitral valve in the sense of regurgitation. Among these, 32 patients presented with hemodynamically insignificant regurgitation, 52 had first-degree mitral valve insufficiency, 6 had second-degree insufficiency, and 2 patients were diagnosed with third-degree mitral valve insufficiency. 56 patients (37.83%) did not show any signs of mitral regurgitation. 72.88% of women and only 55.06% of men were affected by mitral valve insufficiency. The average age of women was 62.71 ± 13.44 years, the average age of men was 56.17 ± 16.12 years. Pearson’s correlation test indicated a weak positive and statistically significant correlation between gender and mitral regurgitation, with a higher prevalence observed in females. As revealed by the regression analysis, this effect was primarily driven by the confounding factor “age”.

Comorbidities such as diabetes mellitus in 24.68%, hypertension in 60.14%, and atrial fibrillation in 12.16%, were determined. Additionally, 25.68% of the patients reported nicotine use. 47.89% of the patients were taking a beta-blocker, 59.86% an ACE inhibitor, and 13.38% an angiotensin II receptor blocker. In total, 16.89% of the patients showed a reduced ejection fraction.

In the overall dataset, correlation between mitral regurgitation and age (*p* < 0.001) and hypertension (*r* = 0.1900, *p* = 0.0208) was significant, whereas diabetes mellitus (*r* = 0.1160, *p* = 0.1602), atrial fibrillation (*r* = 0.1198, *p* = 0.1469), coronary heart disease (*r* = 0.1173, *p* = 0.0739), and nicotine use (*r* = 0.0440, *p* = 0.5958) did not show any significance. In the subgroup of “MR-only”, a significant correlation between mitral regurgitation and atrial fibrillation (*r* = 0.2426, *p* = 0.0462) was found. In the overall dataset, correlation between coronary heart disease and age (*p <* 0.001), nicotine use (*r* = 0.2936, *p* < 0.001), hypertension (*r* = 0.2739, *p* < 0.001), and diabetes mellitus (*r* = 0.1898, *p* = 0.0210) was significant, whereas atrial fibrillation (*r* = 0.0112, *p* = 0.8924) did not show any significance.

Furthermore, the BSA (*p* < 0.001), the length (*p* < 0.001), and internal diameter (*p* < 0.001) of the left ventricle were larger in men (mean BSA = 2.03 m^2^ ± 0.22, mean length = 84.36 mm ± 11.21, mean internal diameter = 54.32 mm ± 9.13) than in women (mean BSA = 1.85 m^2^ ± 0.18, mean length = 75.14 mm ± 7.22, mean internal diameter = 50.15 mm ± 8.02) (Table 1).

**Figure 4:**
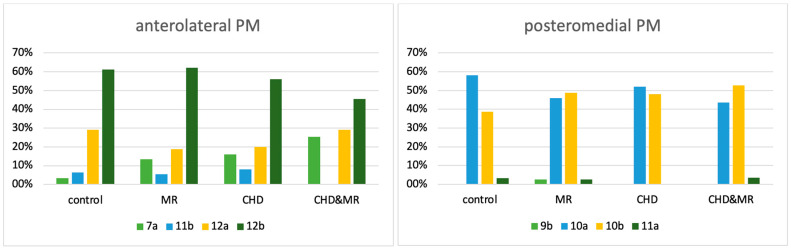
Percentage distribution of papillary muscle position.

A variable distribution of papillary muscle positions was observed in the left ventricle (Table 2, Figure 4). The anterolateral papillary muscle was most frequently located in segment 12b and 12a in all subgroups. The posteromedial papillary muscle was most commonly located in segments 10a and 10b. The most frequent combinations of papillary muscle positioning were observed in the constellations 12b-10a, 12b-10b, 12a-10a, and 12a-10b.

Notably, the presence of the anterolateral papillary muscle in segment 7a was observed in only 3.3% of patients in the control group. In contrast, it was more frequently seen in patients with cardiac disease (MR-only: 13.5%, CHD-only: 16.0%, combined CHD and MR: 25.4%). In the combined CHD and MR subgroup, the association of MR with position 7a was also statistically significant (*p* = 0.0083).

**Figure 5:**
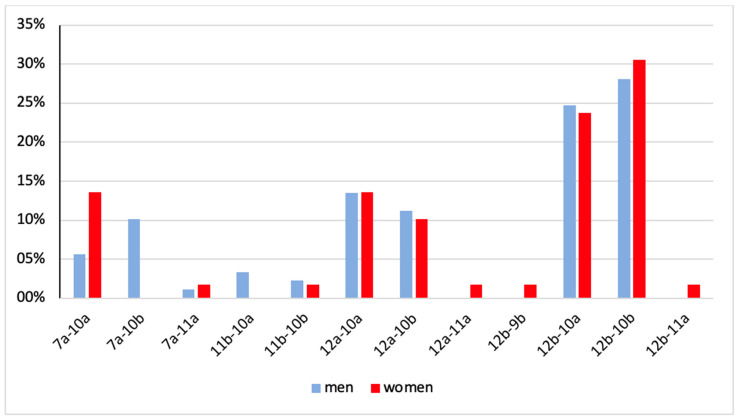
Percentage distribution of papillary muscle positions in men versus women. A clear difference between men and women can be identified for position 7a-10b (10.1% men versus 0.0% women).

No significant sex-based differences in the spatial distribution of papillary muscles were observed within any of the subgroups. However, in the overall dataset, a statistically significant difference was found for position 7a-10b, which occurred in 10.1% of male patients and in no female patients (*p* = 0.0115) (Table 3, Figure 5).

**Figure 6:**
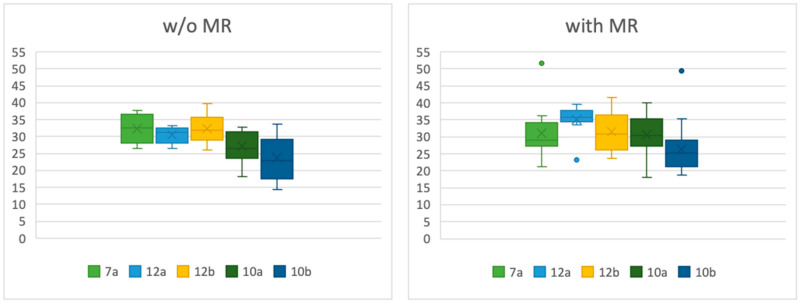
Angles between papillary muscle and ventricular wall in patients without (w/o) MR and with MR in normal-sized ventricles.

**Figure 7:**
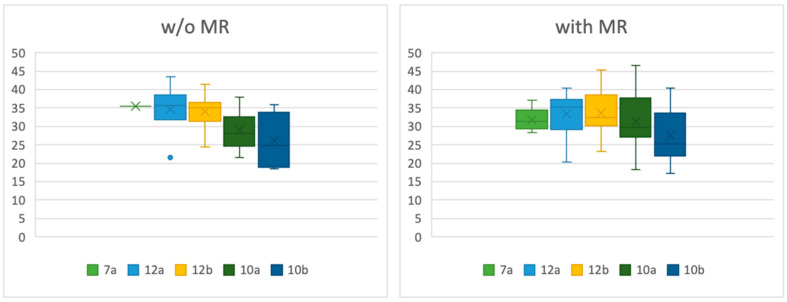
Angles between papillary muscle and ventricular wall in patients without MR and with MR in dilated ventricles.

In the overall dataset, no statistically significant correlation was found between mitral regurgitation and ventricular diameter (*p* = 0.1325) or ventricular length (*p* = 0.1019). In the MR-only subgroup, patients with a normal-sized left ventricle exhibited a significantly larger angle between the papillary muscle and the ventricular wall in position 12a compared to patients without MR (*p* = 0.0339). When considering all patients with MR (overall dataset) versus without MR, those with a normal-sized left ventricle also demonstrated a significantly larger angle in positions 12a (*p* = 0.0095) and 10a (*p* = 0.0460) (Table 4, Figure 6). In the group of dilated ventricles, there was no significant difference between the angles of patients in any group (Table 5, Figure 7).

## 5. Discussion

There are anatomical gender-specific differences in the structure of the human heart, for example, the size of the heart in general [11,12]. The papillary muscle mass, which correlates with left ventricular mass, is generally bigger in men compared to women [13]. Our study confirms the anatomical differences between men and women. The length and internal diameter of the left ventricle were significantly larger in men compared to women.

Berdajs et al. [14] conducted a comprehensive study into various anatomical variations in the papillary muscles themselves; the morphology of the posteromedial papillary muscle exhibited greater variability compared to the anterolateral papillary muscle. Saha and Roy [15] found the anterolateral PM arose mainly from the upper and middle thirds of the sternocostal wall, while the posteromedial PM originated from the middle third of the diaphragmatic wall in 50% of cases, and from the upper or lower third in the remainder. In total, 82% per cent of anterolateral papillary muscles originated from the anterolateral ventricular wall, while 18% arose from the inferior wall. In contrast, the posteromedial papillary muscle was found to originate from the inferior wall in 90% of cases and from the anterolateral region in 10% [16]. Our study identified distinct positional patterns of the papillary muscles. The anterolateral papillary muscles predominantly originated from segments 12b and 12a, corresponding to the anterolateral region of the left ventricular wall, while the posteromedial papillary muscles were primarily associated with segments 10a and 10b, located in the inferior region. In both men and women, the frequency distribution of the papillary muscles in the anterolateral and posteromedial regions closely aligned with the findings of Kavitha et al. [16]. We observed the most frequent combinations of papillary muscle positioning in the constellations 12b-10a, 12b-10b, 12a-10a, and 12a-10b. A difference was found in the combination of 7a-10b (10.1% of men but not in women).

Our study demonstrated a significantly increased occurrence of papillary muscle position 7a in patients with cardiac diseases. Specifically, we identified a statistically significant association between this anatomical variant and the simultaneous occurrence of CHD and mitral regurgitation. Chronic ischemic mitral regurgitation has shown that in the case of ventricular remodeling it can cause papillary muscle displacement and, accordingly, leaflet tethering [17,18]. Li et al. [2] detailed the anatomy and pathology of the left ventricular papillary muscles, emphasizing their crucial role in mitral valve competence because ischemic injury and ventricular remodeling can alter the position and function of the papillary muscle, contributing to MR. The anterolateral papillary muscle benefits from a dual blood supply originating from both the left anterior descending artery (LAD) and the left circumflex artery (LCX) [19]. Therefore, it is assumed that the anterolateral papillary muscle is less sensitive to ischemia than the posteromedial papillary muscle [20], whose blood supply depends on the dominant coronary artery and is provided either by the right coronary artery (right dominant) or the LCX (left dominant) [21]. “Culprit lesions” within the respective coronary risk territories demonstrated a high and comparable predictive value for infarction of the anterolateral and posteromedial papillary muscle. Wendell et al. [22] investigated the coronary tree for “culprit lesions” associated with infarction of the anterolateral and posteromedial papillary muscles. The left anterior descending artery (LAD) emerged as the most frequently occluded vessel. However, posteromedial papillary muscle infarction exhibited a higher prevalence compared to anterolateral papillary muscle infarction. It was therefore concluded that the anatomical location of occlusion plays a central role in determining the occurrence of papillary muscle infarction [22].

Agricola et al. [23] discussed chronic ischemic MR as a complication of myocardial infarction, highlighting that left ventricular remodeling leads to displacement of papillary muscles, which in turn causes leaflet tethering and MR. Uemura et al. [24] reported that ischemic mitral regurgitation is related to leaflet tethering caused by outward displacement of the papillary muscles due to local ventricular remodeling after myocardial infarction. The tethering distance was identified as the main determinant of ischemic MR.

Yiu et al. [17] demonstrated that local left ventricular remodeling—manifested as apical and posterior displacement of the papillary muscles—contributes to excessive mitral valve tenting, independent of global LV remodeling. This may explain why we observed a significantly larger angle in patients with normal-sized ventricles. The increased angles of the papillary muscles in segments 12a and 10a may reflect papillary muscle malposition as a consequence of local left ventricular remodeling. The resulting altered distribution of forces transmitted through the chordae tendineae could, in the absence of compensatory mechanisms, contribute to the development of mitral regurgitation (MR). Since we measured the midventricular diameter at the same height as papillary muscle positioning, we have no corresponding reference values for midventricular diameter. We determined our standard value slightly higher than the standard values described by Stolzmann et al. [9] for measurement in computed tomography. To our knowledge, no previous study has determined papillary muscle position using a segmentation scheme in conjunction with angulation measurements between the papillary muscle and the ventricular wall in a comparable manner. Accordingly, reference values or normative data for this approach are currently not available.

When evaluating the correlations between comorbidities and the presence of mitral regurgitation (MR), no significant association between MR and atrial fibrillation was observed in the overall dataset. However, in the MR-only subset, a significant correlation was identified. This finding may be attributed to the heterogeneity of underlying cardiac conditions in the overall cohort, particularly the coexistence of CHD, which constitutes an independent risk factor for atrial fibrillation [25] and may thereby mask a specific association between mitral regurgitation and atrial fibrillation. An association between hypertension and mitral valve regurgitation was observed. Hypertension is known to promote cardiac remodeling [26], which in turn may influence the position and orientation of the papillary muscles, potentially affecting mitral valve function.

## 6. Clinical Impact

Patients with ventricular remodeling often suffer from secondary mitral valve regurgitation due to papillary muscle displacement [17,18]. Standard annuloplasty showed high rates of recurrent mitral valve regurgitation [27,28,29,30]. A meta-analysis by Harmel et al. [7] revealed better long-term results in patients with secondary MR with restricted leaflet motion during systole (Carpentier Type IIIb [31]) who underwent annuloplasty with subannular repair instead of annuloplasty alone.

Our study indicates that patients with MR but a normal-sized ventricle, with anterolateral papillary muscle located in segment 12a or posteromedial papillary muscle located in segment 10a, exhibit significantly larger angles between the papillary muscle and the ventricular wall. This highlights the importance of position determination and angle measurement as key diagnostic parameters. Despite the absence of global ventricular dilation, an altered papillary muscle position should be anticipated, emphasizing the potential need for subannular repair as part of the surgical treatment strategy. Furthermore, our study identified a significant correlation between an anterolateral papillary muscle positioned in segment 7a and the simultaneous occurrence of CHD and MR. This emphasizes once again that, despite the dual blood supply, the anterolateral papillary muscle is still susceptible to displacement and dysfunction in the context of coronary heart disease and left ventricular remodeling.

Our study highlights the complexity of both the mitral valve apparatus and the clinical entity of mitral regurgitation. In order to gain further diagnostic insights and to expand therapeutic options, additional investigations focusing on the position of the papillary muscles are necessary. Furthermore, not only topographical, but also morphological characteristics play a decisive role in the development of valvular disease [32]. Consequently, the combination of various diagnostic modalities will become increasingly relevant in the future for comprehensive assessment and individualized therapeutic strategies.

## 7. Limitations

Our retrospective study involved 148 non-consecutive patients which were divided into several subgroups. Those subgroups appeared to be small and cannot represent the main population. Furthermore, most patients had a history of cardiac disease, and our control group appeared to be small as well. Nonetheless, the study population was non-homogeneous, comprising patients with various underlying conditions. To understand the physiological position of the papillary muscles, future studies should involve more patients without any history of heart disease.

The echocardiographic data available for analysis were often incomplete, which limited a comprehensive assessment of specific myocardial and valvular pathologies. A detailed examination of patients with severe mitral regurgitation was limited by the small sample size of this subgroup, which restricts the statistical power. The effects of reduced left ventricular ejection fraction and regional wall motion abnormalities were not systematically investigated. To better understand the clinical impact of these factors, future studies should ideally be designed prospectively and include extensive echocardiographic evaluation alongside cardiac computed tomography. Furthermore, conventional coronary angiography should be incorporated to evaluate the impact of coronary heart disease more precisely. Finally, the potential associations between mitral regurgitation, changes in left ventricular ejection fraction, and segmental contractility disturbances warrant further investigation in larger, well-characterized patient cohorts.

Additionally, due to its variable morphology, angle measurement at the posteromedial papillary muscle was more challenging than at the anterolateral papillary muscle. To ensure measurement accuracy, all assessments were performed in close collaboration with experienced radiologists.

## 8. Conclusions

Our study confirms the involvement of subannular structures in the pathogenesis of mitral regurgitation and reaffirms the concept of papillary muscle displacement due to left ventricular remodeling. Both the anterolateral and posteromedial papillary muscles are significantly correlated with the presence of mitral regurgitation, as demonstrated by the increased angles of both papillary muscles and the specific positional changes in the anterolateral papillary muscle. Preoperative diagnostics should include a detailed assessment of all components of the mitral valve apparatus, including papillary muscle position, to enable optimal surgical planning and targeted reconstruction.

## Figures and Tables

**Table 1:** Comparison of body surface area, left midventricular diameter, and left ventricular length between men and women. Both left midventricular diameter and left ventricular length were measured in end-diastole.

	Men				Women				*p*-Value
	Min	Max	Mean	SD	Min	Max	Mean	SD	
body surface area (m^2^)	1.54	2.74	2.03	0.22	1.35	2.14	1.85	0.18	<0.001
midventricular diameter (mm)	29.00	86.70	54.32	9.13	34.9	86.70	50.15	8.02	<0.001
ventricular length (mm)	60.50	131.00	84.36	11.21	58.40	90.20	75.14	7.22	<0.001

**Table 2:** Positions of papillary muscles in the left ventricle. The table on top shows the anterolateral papillary muscle, the table at the bottom the posteromedial papillary muscle.

	Control	MR	CHD	CHD&MR	Total
7a	1	5	4	14	24
11b	2	2	2	0	6
12a	9	7	5	16	37
12b	19	23	14	25	81
total	31	37	25	55	148
	**Control**	**MR**	**CHD**	**CHD&MR**	**Total**
9b	0	1	0	0	1
10a	18	17	13	24	72
10b	12	18	12	29	71
11a	1	1	0	2	4
total	31	37	25	55	148

**Table 3:** Combined positions of the papillary muscles in men, women, and overall.

	7a-10a	7a-10b	7a-11a	11b-10a	11b-10b	12a-10a	12a-10b	12a-11a	12b-9b	12b-10a	12b-10b	12b-11a	Total
men	5 (5.6%)	9 (10.1%)	1 (1.1%)	3 (3.4%)	2 (2.3%)	12 (13.5%)	10 (11.2%)	0 (0.0%)	0 (0.0%)	22 (24.7%)	25 (28.1%)	0 (0.0%)	89 (100%)
women	8 (13.6%)	0 (0.0%)	1 (1.7%)	0 (0.0%)	1 (1.7%)	8 (13.6%)	6 (10.7%)	1 (1.7%)	1 (1.7%)	14 23.7%)	18 (30.5%)	1 (1.7%)	59 (100%)
total	13 (8.8%)	9 (6.1%)	2 (1.4%)	3 (2.0%)	3 (3.0%)	20 (13.5%)	16 (10.8%)	1 (0.1%)	1 (0.1%)	36 (24.3%)	43 (29.1%)	1 (0.1%)	148 (100%)

**Table 4:** Descriptive statistics for angles in patients with MR and without MR, midventricular diameter <52 mm in women and <54 mm in men.

	w/o MI					with MI					
	Min	Max	SD	Mean	Median	Min	Max	SD	Mean	Median	*p*-Value
7a	26.6	37.8	4.58	32.38	32.55	26.6	51.7	7.24	32.04	29.3	0.5135
12a	26.4	33.3	2.53	30.53	31.3	23.2	39.5	4.77	35.04	35.7	**0.0095**
12b	26.0	39.7	4.36	32.33	31.8	23.6	41.5	5.61	31.45	30.8	0.6075
10a	18.3	32.9	4.24	27.08	26.4	18.0	40.1	5.79	30.57	30.35	**0.0459**
10b	14.3	33.6	5.91	23.72	22.9	18.8	49.5	6.77	26.41	25.2	0.6262

**Table 5:** Descriptive statistics for angles in patients with MR and without (w/o) MR, midventricular diameter >52 mm in women and >54 mm in men.

	w/o MI					with MI					
	Min	Max	SD	Mean	Median	Min	Max	SD	Mean	Median	*p*-Value
7a	35.5	35.5	-	35.5	35.5	28.4	37.2	3.10	31.89	31.3	0.2752
12a	21.5	43.5	6.92	34.74	35.7	20.4	40.3	5.65	33.38	35.3	0.6014
12b	24.5	41.5	4.43	33.97	35.1	23.2	45.4	5.67	33.86	32.7	0.8387
10a	21.5	38.0	5.26	29.03	28.1	18.2	46.5	7.30	31.37	29.7	0.3400
10b	18.5	35.8	7.07	36.07	24.9	17.3	40.3	7.1	27.41	25.0	0.5868

## Data Availability

Original data are available on request from the corresponding author.

## Abbreviations

The following abbreviations are used in this manuscript:

CHD	coronary heart disease
CT	cardiac computed tomography
EF	ejection fraction
LAD	left anterior descending artery
LCX	left circumflex artery
MPR	multiplanar reconstruction
MR	mitral regurgitation
PACS	picture archiving and communication system
PM	papillary muscle
w/o	without
